# Supplementary material for: Wood duck nest survival and duckling recruitment is minimally affected by interspecific brood parasitism from hooded mergansers and black-bellied whistling-ducks
Source: PLoS One. 2024 Jul 2;19(7):e0305899. doi: 10.1371/journal.pone.0305899 (PMC11218992; doi:10.1371/journal.pone.0305899)
Supplement: Attachment — Submitted filename: PLOSONE review.pdf [file pone.0305899.s008.pdf]

Review of PONE-D-24-06740
Wood duck nest survival and duckling recruitment is unaffected by interspecific brood parasitism from hooded mergansers and black-bellied whistling ducks.

General Comments:
I am a bit slow on this review, so I'll keep this short, not only in the interest of time, but also because I don't think an extensive review is needed. Overall, this is a solid manuscript that presents some useful information on the impacts of interspecific brood parasitism (IBP) by two species of cavity nesting waterfowl, Hooded Mergansers (HOME) and Black-bellied Whistling-ducks (BBWD), on a third species of cavity nesting waterfowl, the Wood Duck (WODU). There's considerable interest in the ecology, reproduction, and management of Wood Ducks, particularly in terms of nest and hatching success. Managers often use nest boxes to supplement natural cavities, and this has been an important management tool for decades. The potential utility of these practices can be impacted, however, by other species such as HOME and BBWD, who also use these nest boxes and lay their eggs in the nests of Wood Ducks, potentially reducing nest success. Accordingly, managers are concerned about these impacts. Moreover, Black-bellied Whistling-ducks have become increasingly abundant in the southern states as the authors note, and there's a potential for further expansion in the future. A basic study like this provides useful information on the potential impacts of such an expansion.

Beyond the management aspects, there's some interesting evolutionary ecology at play as well. Interspecific brood parasitism has been a focus of much research over the past several decades, particularly on the potential for coevolutionary arms races to arise between hosts and parasites. Most of that research has focused on altricial bird species, such as cuckoos and cowbirds and their hosts. Far fewer studies have examined interspecific parasitism in precocial species such as waterfowl. For precocial birds, the cost of parasitism may be considerably different, and there's a little evidence of co-evolutionary arms races, primarily because the impacts on the host may be much lower. Nonetheless, robust data on these impacts are limited, most of which has come from studies on two species - Canvasbacks and Redhead ducks, with smaller number of studies on Wood Ducks and Hooded Mergansers. Now, with Wood Ducks being exposed to two potential parasitic species, things get a bit more complicated. This is the first study to dive into this three-way interaction. The analyses are robust, and the manuscript is well written. In fact, as I read it, I wondered if the highly sophisticated analysis using Bayesian models were necessary for simple comparison of the effects of IBP on clutch size and hatching success, but they certainly take the level of rigor up a notch. The analysis of recruitment was a useful and novel aspect, although hampered by the small number of 50 returning ducklings (a surprisingly low recruitment rate) and the limited sample of recruits from mixed clutches. Nonetheless, the analysis of recruitment rates relative to initiation date was informative.

I have very few concerns or comments about the manuscript and make only a few small comments and suggestions below. Overall, this paper provides useful information, both from a management perspective and on the impacts of interspecific brood parasitism in precocial species of birds, for which there is a paucity of data.

Specific Comments:

Line 26, 30-31. This is an impressive number of nests, females, and ducklings! A huge effort on the part of the researchers and I just want to highlight the value of data collected over so many nests and over a period of four years. This alone makes the paper stand out among other studies with fewer samples and more limited study periods.

Line 80-82. You might also cite some of Charlotte Roy Nielsen's work on CBP in Wood Ducks - she has published several relevant papers and has done some great work that should be acknowledged.

Nielsen, C. R., P. G. Parker, and R. J. Gates. 2006. Intraspecific nest parasitism of cavity-nesting wood ducks: costs and benefits to hosts and parasites. Animal Behaviour 72:917–926.

Roy Nielsen, C. L., R. J. Gates, and P. G. Parker. 2006. Intraspecific nest parasitism of wood ducks in natural cavities: comparisons with nest boxes. The Journal of Wildlife Management 70:835–843.

Roy Nielsen, C. L., P. G. Parker, and R. J. Gates. 2008. Partial clutch predation, dilution of predation risk, and the evolution of intraspecific nest parasitism. The Auk 125:679–686.

Line 149. I recognize the challenges of visiting so many nest boxes on a regular interval. A weekly check (~7d) is typical of these kinds of studies, but I do wonder whether that generates enough encounters to provide sufficient data for a full-on DSR survival analysis - effectively 2-3 nest checks (encounters) per site?

Lines 211-221. I am, admittedly, a statistical dinosaur, and I very much appreciate the rigor and contemporary approach to these analyses. Using Bayesian multilevel Poisson models to estimate the effects of clutch type (HOME, BBWD parasitism) on clutch size is undoubtedly powerful, but I do wonder whether this is absolutely necessary. Is it akin to using a sledge hammer to simply drive a nail? I realize the value and strengths of Bayesian analyses and they are the new standard, but it does seem like a very sophisticated analysis for what should be a quite simple comparison of clutch size as a function of clutch type. I don't mean this as a criticism, but as an old-timer, I do sense a growing tendency to use the newest techniques simply because they are the newest technique, rather than one that's absolutely necessary. I don’t' disagree with doing things correctly and with the best possible method - perhaps the authors might make a short statement about their overall statistical approach and why it was chosen?

Line 221. So, hatch success was only considered for successful nests, which makes sense because including failed nests would conflate both nest success and hatch success, and would have an excess of 0's, leading to a very zero-inflated distribution. The authors might want to point out that in effect, they are undertaking a hierarchical approach to: (1) first assess the effect on nest success. If the nest is successful, then (2) assess the factors influencing clutch size and hatch success, and (3) then, with the sample of recruits, assess duckling survival and the effects of initiation date (not parasitism) on duckling recruitment. This may not be necessary but it would help a reader follow the inherent hierarchical framework and make it explicit.

Line 260-262. How did you remove/exclude males? Did you simply just split the sample of ducklings in half or did you actually sex ducklings (possible to do, but difficult and time consuming).

You should also clarify whether your recruits include ducklings that were "recaptured" by RFID reads, rather than being caught on the nest. That was never made clear (i.e., the hidden recruits - we have observed these females comprising a large fraction of the recruits in our own wood duck studies).

Line 281-282. Why is the entry for the transition from "duckling" in state $_t$ to "seen as adult" in state $_{t+1}$ "0"in the observation matrix? I am probably not understanding how this analysis is done; naively I would think that entry would be the observed probability of marked ducklings that were then caught as adults (Φ_d). If not, how the is Φ_d) estimated (lines 288-290)? Maybe a bit more description for readers unfamiliar with this method.

Line 301-304. I am not sure that using "nest initiation date as replacement variable to represent the temporal variation in brood composition" really accounts for the effects of parasitism. Certainly, there is temporal overlap in the timing of early Wood Duck nests and Hooded Merganser parasitism, and late Wood Duck nests and Black-bellied Whistling-duck parasitism, but this is confounded by other seasonal changes in brood habitat, etc. that may have a strong influence on both mixed and non-mixed clutches. The question is whether, given (or controlling for) those seasonal patterns, there an additional influence of parasitism? I understand that small sample sizes of recruits for mixed clutches prevent such a contrast, but I don’t think using "initiation date" is valid "replacement variable". You simply are assessing the effect of initiation date (which is useful) but can infer little about the effect of parasitism per se.

Line 391-394. The percentages reported here refer to the proportions of the 50 recaptured ducklings, but that's not relevant given that different numbers of ducklings were tagged from each type of clutch. More useful would be the number of recaptured ducklings from each clutch type relative to the number that were tagged for that clutch type. So, 6 of 186 (3.25%) ducklings returned from mixed HOME clutches, 30 of 1389 (2.16%) returned from early WODU nests, 1 of 224 (0.45%) from mixed BBWD clutches, and 13 of 666 (1.98%) from late WODU clutches. That better represents the small number of recruits and the similarity among clutch types. The proportion of the 50 is meaningless given the different numbers tagged.

Line 412-416. This is a reasonable explanation, but might the lower survival of mixed merganser clutches also be due to aggressive interference among merganser and wood duck females, or perhaps just the effects of large rounder eggs of mergansers impacting wood duck incubation or nest success? Moreover, if this pattern were simply due to earlier nests including more "dump nests that are never incubated", that should be true for non-mixed early clutches as well and so would not explain the lower DSR of mixed merganser clutches. I think a more discussion of the reduced success in mixed merganser clutches is warranted.

Line 418-420. Wasn't the second term of the quadratic relationship with clutch size negative (line 377), such that DSR declines at larger clutch sizes? That also seems to be apparent in Figure 4. It appears you were interpreting this as indicating an asymptote, but it could be

(and appears to be) a declining function after 30-35 eggs (you did not show the predicted function for larger clutch sizes but it trends down). Other studies of wood ducks (e.g. Semel and Sherman) found a decline in hatch success when clutch sizes become extreme. You might want to be more cautious about suggesting that there was "no evidence that excessive clutch sizes diminished survival".

Line 424-425. Again, a bit of caution is warranted here. Given that DSR is lower in the mixed merganser clutches, I would be cautious about suggesting the parasitic egg laying and larger clutch sizes can be a protective mechanism against nest abandonment.

Line 430-432. This is an interesting idea that over 25 years there has been adaptation to excessive brood parasitism. It's not clear how this might arise and without any evidence from other studies to support such a mechanism, perhaps this is too speculative? Admittedly, I am one who loves to speculate rampantly (and am often criticized for it), but I think you need a bit more evidence or at least a plausible mechanism for there to be support for this suggestion.

Overall, I enjoyed reading this manuscript and believe it is a very useful contribution. The sample sizes are extensive, the analyses are thorough and robust (even given my comments about using more sophisticate analytical methods than might be necessary), and there is a need for more studies such as this on precocial species if we are to fully understand the ecological, evolutionary, and management implications of IBP in birds.

I hope my comments are useful.

John Eadie
UC Davis
