# Supplementary material for: Wood duck nest survival and duckling recruitment is minimally affected by interspecific brood parasitism from hooded mergansers and black-bellied whistling-ducks
Source: PLoS One. 2024 Jul 2;19(7):e0305899. doi: 10.1371/journal.pone.0305899 (PMC11218992; doi:10.1371/journal.pone.0305899)
Supplement: Attachment — Submitted filename: ResponseToReviewers.docx [file pone.0305899.s010.docx]

-Author’s responses to comments shown in red text.

**Editor Comments**

**ABSTRACT**

**INTRODUCTION**

**METHODS**

Lines 235-236 and line 243:  Considering that epsilon is typically used to symbolize unexplained error, as opposed to the variance explained by random effects, I would urge you to replace it with “site ID” in this model formula, and then to remove your parenthetical reference to epsilon on line 243.  This approach would also require removing the definition of epsilon on line 236.

-Done!

Lines 289 and 293:  Following the same logic, replace the epsilon with “brood ID” on line 289 and delete the parenthetical epsilon on line 293.

-Done!

Line 297:  I think you were better off with the language you had in place previously, although I would suggest adding a brief explanation of why the standardization was necessary, similar to what you provided in your response to reviewers.

-Done! This now reads: “Prior to running our models, we scaled all variables to have a mean of 0 and a standard deviation of 1. This process places all covariates on the same scale, facilitating the comparison of beta estimates across different covariates [54].”

**RESULTS**

**DISCUSSION**

**FIGURES**

Fig. 1:  Please limit this figure to three panels, one for mixed HOME, one for mixed BBWD, and one for non-IBP nests.  By separating the latter into early and late phases of the nesting period, you’re placing a time-constraint on each distribution that doesn’t naturally exist.  Creating one distribution for the non-parasitized nests will presumably create the sort of bimodal distribution Dr. Rohwer is alluding to in his comments, and even if it doesn’t it won’t invalidate your approach to dividing up the nesting season into two distinct periods for the purposes of statistical analysis.

-Done!

Fig. 4:  Please remove “Observed nests” from the figure and move “Recruited female ducklings” to the y-axis so that it reads “Density of recruited female ducklings.”  Also, clarify in the accompanying caption that these data consider females only.

-Done! Given the distribution of nest initiation dates for all the nests we observed throughout the study, which was moved to Fig 1 based on the previous comment, we deleted the top plot from this figure and left everything else the same. We are happy to modify this figure further if needed 😊
